# Information and Divergence Measures

**DOI:** 10.3390/e25040683

**Published:** 2023-04-19

**Authors:** Alex Karagrigoriou, Andreas Makrides

**Affiliations:** 1 Laboratory of Statistics and Data Analysis, Department of Statistics and Actuarial-Financial Mathematics, University of the Aegean, GR-83200 Karlovasi, Greece; makrides.an@unic.ac.cy; 2 Department of Computer Science, University of Nicosia, CY-1700 Nicosia, Cyprus

The present Special Issue of *Entropy*, entitled Information and Divergence Measures, covers various aspects and applications in the general area of Information and Divergence Measures.

Measures of information appear everywhere in probability and statistics. They play a fundamental role in communication theory. They have a long history dating back to the papers of Fisher, Shannon, and Kullback. There are many measures each claiming to capture the concept of information or simply being measures of divergence or distance between two probability distributions. Numerous generalizations of such measures also exist. 

The concept of distance is important in establishing the degree of similarity and/or closeness between functions, populations, and distributions. The intense engagement of many authors with entropy and divergence measures demonstrates the significant role they are playing in the sciences. Indeed, distances and entropies are related to inferential statistics, including both estimation and hypothesis testing problems [1,2,3,4,5,6], model selection criteria [7,8,9] and probabilistic and statistical modelling with applications in multivariate analysis, actuarial science, portfolio optimization, survival analysis, reliability theory, change-point problems, etc. [10,11,12,13,14,15]. Thus, the significance of entropy and divergence measures that emerges in these and many more scientific fields is a topic of great interest to scientists, researchers, medical experts, engineers, industrial managers, computer experts, data analysts, etc.

All the articles included in this Special Issue were reviewed and accepted for publication because they have been found to contribute research works of the highest quality and at the same time, they highlight the diversity of the topics in this scientific area. The issue presents twelve original contributions that span a wide range of topics. In [16], the authors demonstrate how to employ the techniques of the calculus of variations with a variable endpoint to search for the closest distribution from a family of distributions generated via a constraint set on the parameter manifold. In [17], the authors consider weighted Tsallis and Kaniadakis divergences and establish inequalities between these measures and Tsallis and Kaniadakis logarithms. In [18], LPI waveforms are designed within the constraints of the detection performance metrics of radar and PISs, both of which are measured by the Kullback–Leibler divergence, and the resolution performance metric, measured by joint entropy with the solution based on the sequential quadratic programming method. In [19], a bootstrap approximation of the Kullback–Leibler discrepancy is utilized to estimate the probability that the fitted null model is closer to the underlying generating model than the fitted alternative model. The authors also propose a bias correction either by adding a bootstrap-based correction or by adding the number of parameters in the candidate model. In [20], the authors extend, and compute information measures related to Shannon and Tsallis entropies, for the concomitants of the generalized order statistics from the Farlie–Gumbel–Morgenstern family. In [21], the evaluation of academic performance by using the statistical K-means (SKM) algorithm to produce clusters is investigated. A simulation experiment on the top 20 universities in China shows the advantages of the SKM algorithm over traditional methods. In [22], the authors introduce a closed-form expression for the Kullback–Leibler divergence between two central multivariate Cauchy distributions used in different signal and image processing applications where non-Gaussian models are needed. In [23], restricted minimum Rényi’s pseudodistance estimators are defined, and their asymptotic distribution and influence function are derived. Further, robust Rao-type and divergence-type tests based on minimum Rényi’s pseudodistance and restricted minimum Rényi’s pseudodistance estimators are considered, and their asymptotic properties are obtained. In [24], a skew logistic distribution is proposed and extended to the skew bi-logistic distribution to allow the modelling of multiple waves in epidemic time series data. The proposed distribution is validated by COVID-19 data from the UK and is evaluated for goodness-of-fit using the empirical survival Jensen–Shannon divergence and the Kolmogorov–Smirnov two-sample test statistic. In [25], an approach for the derivation of families of inequalities for set functions is suggested and applied to obtain information inequalities with Shannon information measures that satisfy sub/supermodularity and monotonicity properties. The author also applies the generalized Han’s inequality to analyse a problem in extremal graph theory, with an information–theoretic proof and interpretation. In [26], the authors focus on a general family of measures of divergence and purpose a restricted minimum divergence estimator under constraints and a new double-index (dual) divergence test statistic which is thoroughly examined. Finally, in [27], by calculating the Kullback–Leibler divergence between two probability measures belonging to different exponential families dominated by the same measure, the authors obtain a formula that generalizes the ordinary Fenchel–Young divergence and define the duo Fenchel–Young divergence which is equivalent to a duo Bregman divergence. The author also proves that the skewed Bhattacharyya distances between truncated exponential families amount to equivalent skewed duo Jensen divergences.
